# Improving insulin sensitivity, liver steatosis and fibrosis in type 2 diabetes by a food-based digital education-assisted lifestyle intervention program: a feasibility study

**DOI:** 10.1007/s00394-021-02521-3

**Published:** 2021-04-11

**Authors:** Oana P. Zaharia, Yuliya Kupriyanova, Yanislava Karusheva, Daniel F. Markgraf, Konstantinos Kantartzis, Andreas L. Birkenfeld, Michael Trenell, Aarti Sahasranaman, Chris Cheyette, Theresa Kössler, Kálmán Bódis, Volker Burkart, Jong-Hee Hwang, Michael Roden, Julia Szendroedi, Dominik H. Pesta

**Affiliations:** 1 Institute for Clinical Diabetology, German Diabetes Center, Leibniz Center for Diabetes Research at Heinrich Heine University, Düsseldorf, Germany; 2 German Center for Diabetes Research (DZD), München-Neuherberg, Germany; 3 Department of Internal Medicine, Division of Diabetology, Endocrinology, and Nephrology and Institute of Diabetes Research and Metabolic Diseases (IDM), University Hospital Tübingen, Tübingen, Germany; 4 NIHR Innovation Observatory, Newcastle University, Newcastle Upon Tyne, UK; 5 Changing Health, Newcastle Upon Tyne, UK; 6 Carbs and Cals Ltd, London, UK; 7 Division of Endocrinology and Diabetology, Medical Faculty, Heinrich Heine University, c/o Auf‘m Hennekamp 65, 40225 Düsseldorf, Germany

**Keywords:** Digital education, Type 2 diabetes, Diabetes management, Insulin sensitivity, Non-alcoholic fatty liver disease

## Abstract

**Purpose:**

Recent trials demonstrated remission of type 2 diabetes and non-alcoholic fatty liver disease (NAFLD) following formula diet-induced weight loss. To improve the outreach for populations in need, many mobile health apps targeting weight loss have been developed with limited scientific evaluation of these apps. The present feasibility study investigated the effects of a novel approach incorporating a regular ‘whole food-based’ low-calorie diet combined with app-based digital education and behavioral change program on glucose metabolism and disease management.

**Methods:**

Twenty-four individuals with type 2 diabetes followed this approach supported by weekly coaching calls for 12 weeks. Phenotyping included bioimpedance analysis, mixed-meal tolerance test, magnetic resonance spectroscopy and transient elastography for assessing liver fat content and liver stiffness.

**Results:**

Over 12 weeks, participants reduced their body weight by 9% (97 ± 13 to 88 ± 12 kg), body mass index (BMI; 33 ± 5 to 29 ± 4 kg/m^2^), total fat mass (31 ± 10 to 27 ± 10%) (all *p* < 0.01) and liver fat by 50% alongside with decreased liver stiffness. Target HbA1c (< 6.5%) was achieved by 38% and resolution of NAFLD (liver fat content < 5.6%) was observed in 30% of the participants.

**Conclusion:**

This novel approach combining digital education with a low-calorie diet results in effective improvements of body weight, glycemic control and NAFLD and could complement existing care for patients with type 2 diabetes.

**Trial registration:**

NCT04509245

## Introduction

Increased consumption of energy-dense foods, obesity and non-alcoholic fatty liver disease (NAFLD), characterized by hepatocellular lipid content (HCL) of more than 5.6%, are the major metabolic drivers for development of type 2 diabetes [1–3]. Risk of NAFLD progression is higher in patients with type 2 diabetes, which in turn increases other diabetes-related complications, such as cardiovascular morbidity and mortality [4]. Moderate weight loss can halt the vicious cycle by normalizing hepatic steatosis, insulin resistance and hyperglycemia [5]. This underlines the clinical relevance of intensive dietary management also in NAFLD [6]. Current nutritional treatment guidelines for adults with type 2 diabetes focus on improving glycemic control, body weight management and cardiovascular risk factors. Although these guidelines emphasize individualized nutritional therapy, recommendations generally promote healthy whole foods with high fiber content and low added sugars and refined grains [7, 8]. With regard to NAFLD, dietary recommendations mostly follow guidelines for adults with obesity and/or type 2 diabetes with a focus on energy restriction and diet quality [9, 10]. Along these lines, the Mediterranean diet has been suggested as a successful lifestyle recommendation for NAFLD [11]. Dietary energy restriction by complete food replacement via formula diets is also able to induce diabetes remission and normalization of HCL in a subset of patients [12, 13]. However, it remains unknown whether similar effects can be achieved with a regular, non-formula based diet in connection with digital health education and coaching. Latest obesity management guidelines advocate for the implementation of emerging technologies and virtual medicine incorporating online education, coaching as well as weight and activity tracking to improve weight-loss outcomes [14]. Although numerous applications are available for weight loss and diabetes management and mobile health interventions hold great promise to optimize treatment strategies for several chronic diseases, many studies using such applications (apps) are of insufficient quality [15, 16]. There is also room for improvement as the functionality of some apps is limited and app development is rarely evidence-based [17]. Currently, it is unclear whether stand-alone use of mobile health apps is sufficient or whether adjuncts, such as phone counselling, should be used concomitantly to improve their efficacy for body weight management. To take account of these novel developments and challenges derived thereof, the aim of this feasibility study was to assess whether a combined approach using a ‘real food-based’ low-calorie diet in connection with a smartphone app and weekly coaching calls for patients with diabetes results in improved body weight, metabolism and disease management.

## Subjects and methods

### Participants

**Fig. 1:**
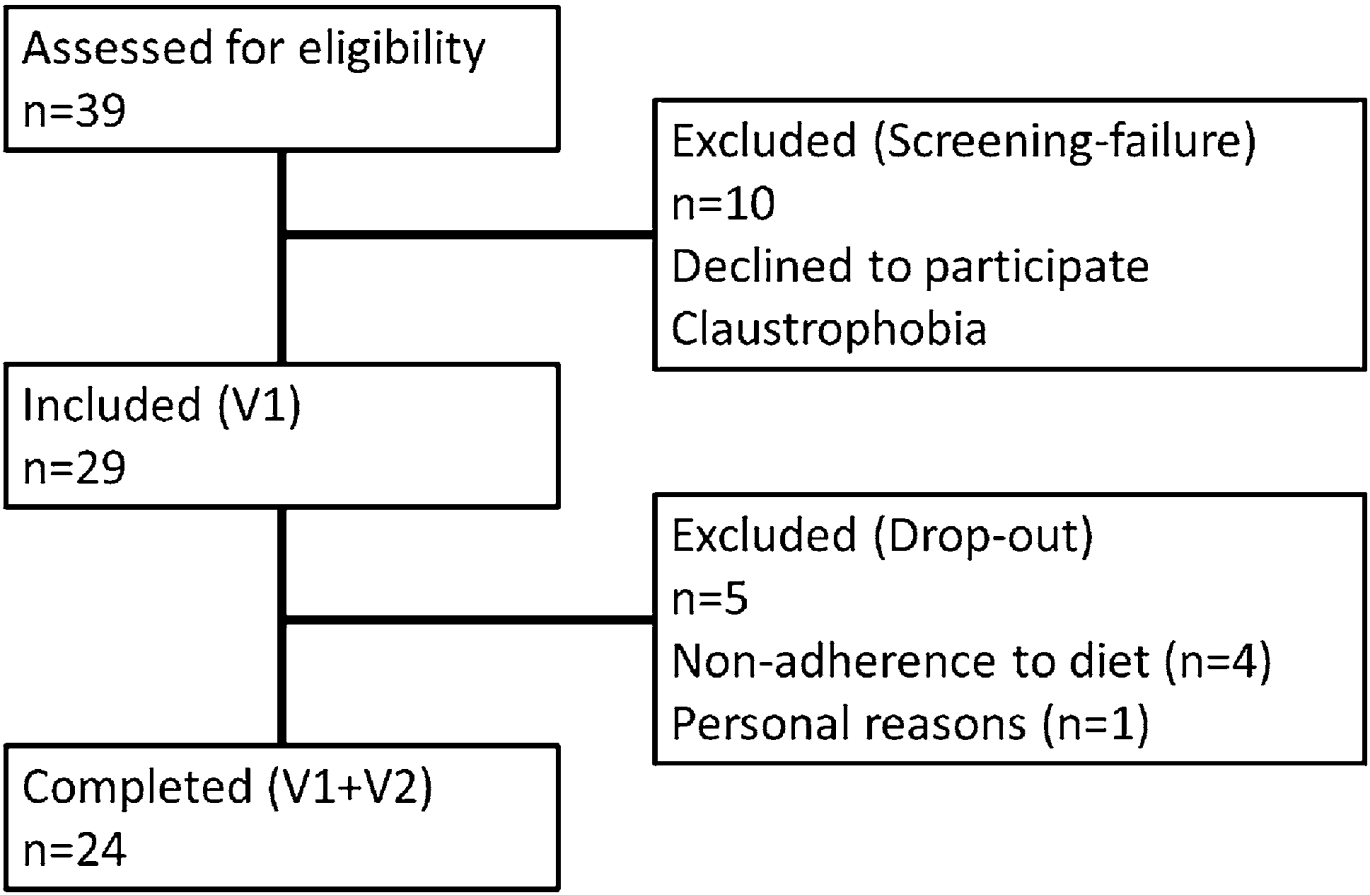
Recruitment flow diagram

We included 24 patients with type 2 diabetes from the German Diabetes Study (GDS) with less than 4 years disease duration and not receiving insulin. Participants were recruited between 10/2018 and 07/2019 and were included after giving informed consent to the study protocols [18] (Fig. 1).

#### Intervention

Before the start of the intervention, participants documented their dietary intake by filing a food diary for 3 days. During the 12-week intervention, participants were asked to adhere to a real food-based low-calorie diet supported by an app-guided digital education program as well as a low-calorie recipe book [19] and received weekly coaching calls by specifically trained nutritionists. The recipe book provides a variety of individual meals and their caloric content and serves as a guide for low-calorie meal choices for the participants [19]. Patients were instructed to adhere to a balanced low-calorie diet (average 850 kcal/day) consisting mainly of high-protein foods with low glycemic index of their choice. Throughout the study, participants photographically documented every food item they consumed. These images were acquired by smartphone through the app and uploaded to an online portal, where they could be accessed and evaluated by the coaches. Based on these images, portion size as well as food items and caloric intake was estimated by trained nutritionists by randomly choosing two days throughout the study. Habitual food intake prior to study inclusion was estimated using 3-day dietary protocols and data were analyzed using the Prodi system (Prodi 6.3.0.1, Nutri-Science GmbH). During the weekly coaching calls, trained coaches provided structured behavior change and motivated the participants to follow a healthy diet, photographically document their daily dietary intake and tracking their body weight, at least on a weekly basis, via the app.

### Digital health application

The Changing Health App (Changing Health, Newcastle, UK) was designed specifically for type 2 diabetes and has been used before in pilot studies in the United Kingdom and the Netherlands. The content of the app also underwent medical and ethical review (ethics boards Heinrich-Heine-University Düsseldorf, reference number 4508). At first visit, patients installed the app and completed an app-based digital education program, consisting of type 2 diabetes education and behavioral change accompanied by weight tracking and dietary monitoring. Patients used the app throughout the study and adherence was monitored  based on logins. Participants were advised not to change their physical activity behavior during the intervention. To monitor their activity, daily steps were recorded via the app. Glucose lowering medication was paused three days prior to metabolic tests.

### Metabolic testing

Comprehensive phenotyping before and after the intervention after an overnight fast included a mixed-meal tolerance test (MMTT; Resource Protein, Nestlé Health Science) yielding the oral glucose insulin sensitivity (OGIS) index. The MMTT provides an estimate of endogenous insulin secretion and insulin sensitivity [20]. The ingested drink mimics a conventional meal delivering 463 kcal (macronutrient composition: 13 g fat, 52 g carbohydrates (30 g sugar) and 35 g protein). Blood samples for MMTT were taken every 30 min over 180 min for measuring glucose, insulin and C-peptide [18]. Fat mass and fat-free mass were assessed by bioimpedance analysis (BioElectrical Impedance Analyzer System, RJL Systems, Detroit, MI, USA). Proton magnetic resonance spectroscopy was performed on a 3-T MR scanner (Achieva X-series, Philips Healthcare) for measurement of HCL expressed as % to water signal [21]. FibroScan (Roll-stand 530, Echosens, Paris, France) was performed by a trained physician using a minimum of 10 valid measurements (interquartile range < 0.30) for liver stiffness (E) and Controlled Attenuation Parameter (CAP) to assess the degree of steatosis [22].

### Ethics

The study is approved by local institutional review boards (ethics boards Heinrich-Heine-University Düsseldorf, reference number 4508, amendment 20, and University Hospital Tübingen reference number 385/2018BO2). This trial was retrospectively registered with ClinicalTrials.gov Identifier: NCT04509245 and was performed according to the Declaration of Helsinki.

### Statistics

Data are presented as mean ± standard deviation. *P*-values < 0.05 were considered to indicate statistically significant differences. Pearson correlation coefficient was used to assess associations between metabolic parameters. Paired t-tests were used to assess differences between baseline and post-intervention follow-up in the study population. Normal distribution was assessed by Shapiro–Wilk test. Assuming a baseline HCL of 8.6 ± 8.2 (mean ± SD) [23] power calculations estimated reaching a *β* of 90% by including 24 participants if the true HCL reduction would equal 7 percentage points. Statistical analyses were performed with SAS (version 9.4; SAS Institute, Cary, USA) and GraphPadPrism (version 7.03; GraphPad Software, San Diego, USA).

## Results

**Fig. 2:**
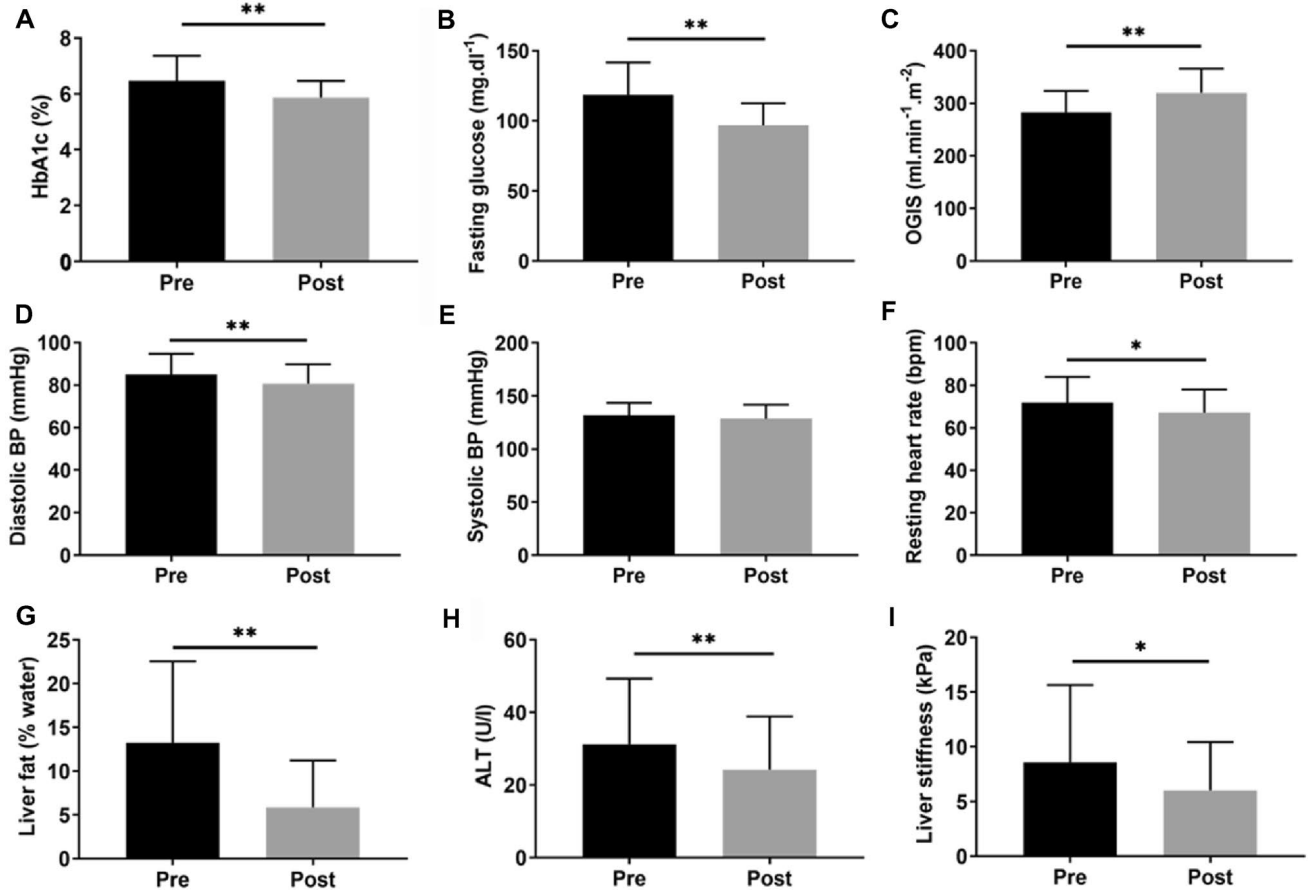
Metabolic parameters before and after the intervention. Glycemic control (**a**, **b**) and insulin sensitivity (**c**), cardiovascular parameters: blood pressure (**d**, **e**) and resting heart rate (**f**) and liver parameters: HCL (**g**), ALT (**h**) and stiffness (**i**). Black bars represent baseline data while post-intervention data are shown as grey bars. *p < 0.05; **p < 0.01. Significant differences as determined by paired samples *t*-test for pre-post comparison. *ALT* alanine aminotransferase, *BP* blood pressure, *HbA1c* glycated hemoglobin, *OGIS* oral glucose insulin sensitivity index

**Fig. 3:**
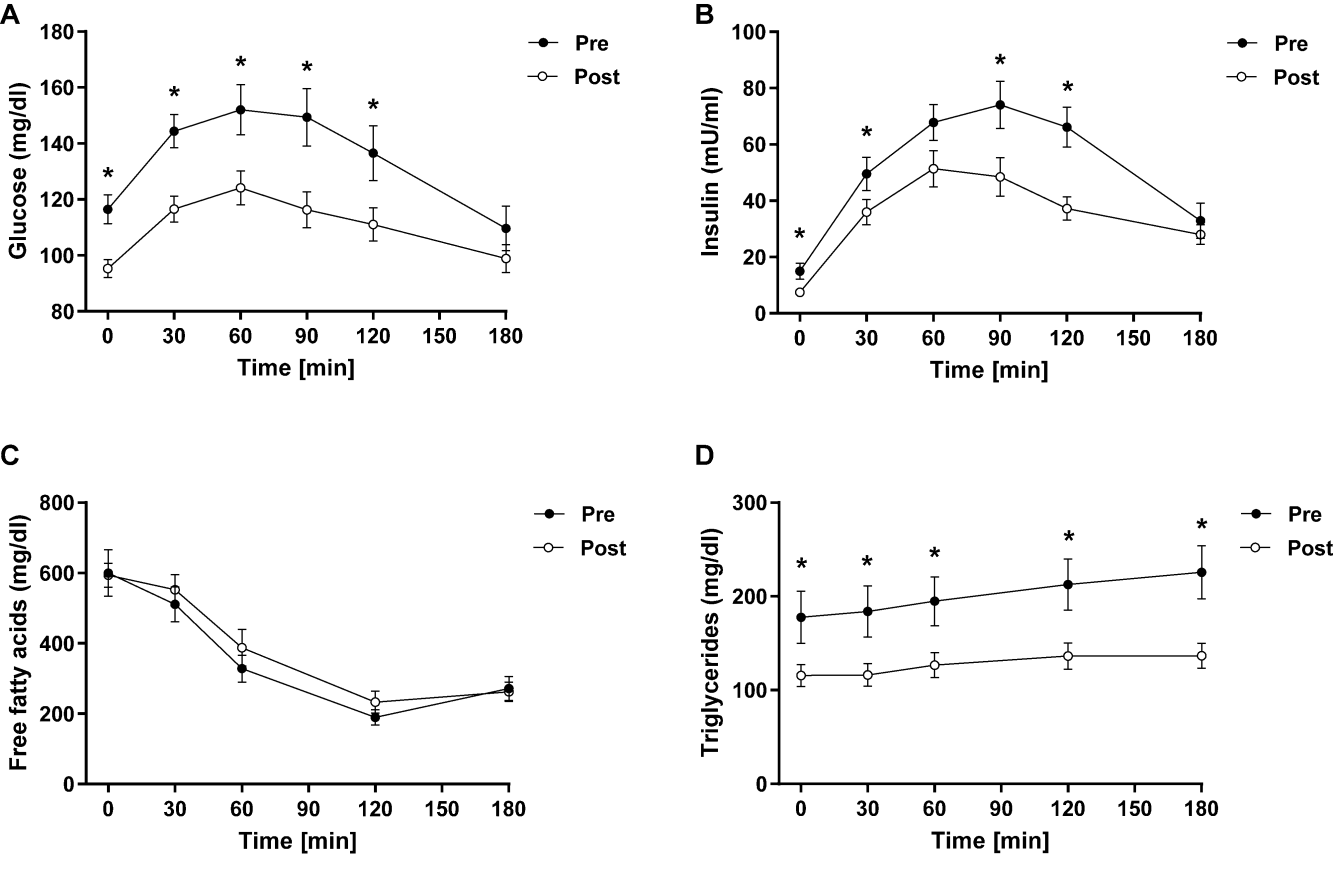
Metabolic response to mixed-meal tolerance test before and after intervention. Metabolic parameters during the mixed-meal test before and after the intervention: glycemia (**a**), insulin secretion (**b**), free fatty acids (**c**) and triglycerides (**d**). Black dots and respective error bars represent baseline data while post-intervention data are shown as white dots and respective error bars. *p < 0.05. Significant differences as determined by paired samples *t*-test for pre-post comparison

Main anthropometric and metabolic parameters are presented in Table 1. In brief, the present study included 14 female and 10 male participants with type 2 diabetes and a mean age of 56 ± 8 years. Participants decreased their body weight by 9% from 97 ± 14 to 88 ± 12 kg, BMI from 33 ± 5 to 29 ± 4 kg/m^2^ and fat mass from 31 ± 10 to 27 ± 10% (all *p* < 0.01). Caloric intake decreased on average from 1753 ± 408 to 868 ± 108 kcal/day (*p* < 0.01). The approximate macronutrient composition of the diet was roughly 40% energy from fat, 28% energy from protein and 32% energy from carbohydrates. Participants also improved their glycemic control, fasting glucose and OGIS (Fig. 2a–c). Similar results were observed for metabolic changes during MMTT (Fig. 3). In brief, the area under the curve for circulating glucose, insulin and triglycerides during the 180 min of the MMTT was lower post-intervention compared to study inclusion (all *p* < 0.05). There was no change in plasma free fatty acid levels during the MMTT.

**Table 1:** Baseline and follow-up characteristics of patients with type 2 diabetes (*n* = 24)

Parameter	Baseline	Follow-up	Change (%)	*p* value
Age (years)	56 ± 8	56 ± 8	–	–
Sex (% female)	58	58	–	–
Body weight (kg)	97.0 ± 13.9	87.7 ± 12.1	− 10	< 0.01
BMI (kg.m^−2^)	32.6 ± 4.6	29.4 ± 3.9	− 10	< 0.01
Fat mass (%)	30.9 ± 9.5	27.4 ± 10.2	− 11	< 0.01
Fasting C-peptide (ng.ml^−1^)	3.3 ± 1.7	2.5 ± 1.0	− 24	< 0.05
Insulin AUC MMTT (a.u.)	6969 ± 3899	4868 ± 2398	− 30	< 0.05
Total cholesterol (mg.dl^−1^)	185.9 ± 42.0	174.4 ± 40.6	− 6	0.09
LDL-cholesterol (mg.dl^−1^)	116.9 ± 39.6	109.6 ± 40.4	− 6	0.23
HDL-cholesterol (mg.dl^−1^)	48.2 ± 14.0	50.4 ± 11.7	+ 5	0.09
Fasting triglycerides (mg.dl^−1^)	197.0 ± 127.8	122.7 ± 60.8	− 38	< 0.01
AST (U.l^−1^)	25.5 ± 11.4	23.3 ± 9.5	− 9	0.21
GGT (U.l^−1^)	53.3 ± 72.2	36.5 ± 47.1	− 32	< 0.05
hsCRP (mg.dl^−1^)	0.4 ± 0.5	0.6 ± 1.0	+ 50	0.33
TSH (µIE.ml^−1^)	1.9 ± 1.1	1.3 ± 0.9	− 32	< 0.05
CAP (dB.m^−1^)	326 ± 64	263 ± 56	− 19	< 0.01

Data are given as mean ± standard deviation or percentages, paired samples *t*-test was used for pre-post comparison

*AST* aspartate aminotransferase, *AUC* area under the curve, *BMI* body mass index, *CAP* controlled attenuation parameter, *GGT* gamma glutamyl-transferase, *HDL* high-density lipoproteins, *hsCRP* high-sensitivity C-reactive protein, *LDL* low-density lipoproteins, *MMTT* mixed-meal tolerance test, *TSH* thyroid-stimulating hormone

Significant differences as determined by  paired samples *t*-test

Diastolic, but not systolic blood pressure decreased alongside with a reduction in resting heart rate (Fig. 2d–f). HCL, alanine aminotransferase activity and liver stiffness also decreased with the intervention (Fig. 2g–i). Changes in HCL correlated with alterations in fasting glucose (*r* = 0.56, *p* = 0.006), changes in HbA1c (*r* = 0.59, *p* = 0.003) and baseline fasting glucose (*r* = 0.41, *p* = 0.04) but did not correlate with changes in body weight or fat mass. No hypoglycemic episodes or other adverse events were reported during the intervention. The number of steps taken by the participants during the intervention remained constant and equaled on average ~ 7000 steps/day.

## Discussion

This feasibility study demonstrates that a combined approach utilizing a ‘real food’-based low-calorie diet, supported by digital tools, is viable and yields significant changes in body weight, liver health and glucose metabolism in people with type 2 diabetes. On average, one in three participants normalized their HbA1c and HCL content over a 12 week period. This approach offers an alternative to meal replacements to achieve type 2 diabetes remission. Recent data demonstrated that significant weight loss (> 10%) is associated with a restoration of pancreatic function and normalization of HbA1c [12]. In these studies, weight loss was achieved using total dietary replacement, targeting 850 kcal/day over a 12-week period [12, 13]. Whilst clinically effective, costs and logistics of supplying meal replacements for every meal for a patient over a 12-week period may be prohibitive. Here, we demonstrate the feasibility of a ‘real food’-based low-calorie approach, supported by digital tools, in achieving a 10% weight loss and accompanying metabolic benefits. According to guidelines, people with type 2 diabetes should aim at > 5% weight loss by intensive lifestyle interventions [24]. With the present approach, nine in ten participants (90%) achieved or surpassed this goal with one in three (38%) reaching target HbA1c (criterion < 6.5%). Our population was not yet on insulin therapy, which made it safe and feasible to implement a low-calorie diet without changes of medication throughout the study, thus minimizing hypoglycemia risk.

Smartphone applications to supported health interventions are promising tools for individuals with diabetes, likely improving diabetes care and disease self-management [25]. We extend the use of this novel approach in patients with diabetes-related NAFLD.

Excessive caloric intake rich in saturated fats, refined carbohydrates and/or sugar-sweetened beverages, has been implicated in the development of NAFLD [26–28] to occur via increased HCL [3, 26]. But diets containing saturated fat, not only promote hepatic triglyceride accumulation, but also favor gluconeogenesis, resulting from allosteric activation of pyruvate carboxylase flux and increased glycerol flux, and induce insulin resistance by lipid-mediated inhibition of insulin signaling [2]. Consequently, lower caloric intake and improved nutrient quality shall reduce HCL, as already described for Mediterranean diets [11]. The Mediterranean diet is characterized by reduced carbohydrate—particularly sugars and refined carbohydrates—and saturated fat content and increased monounsaturated and omega-3 fatty acid content. Body weight loss of 10% is necessary to improve or reverse NAFLD [1]. Negative energy balance was induced via dietary improvements monitored by coaches based on logged dietary intake data from participants. This likely contributed to the relevant loss of HCL, associating improved glucose metabolism, insulin sensitivity and liver function [13]. Of note, loss of body weight did not associate with HCL reductions. Within this time frame, other factors, such as decreasing glucotoxicity and hyperinsulinemia, may affect reductions in HCL in patients with type 2 diabetes. Decreased fasting triglyceride levels likely aided improved insulin sensitivity. Similarly, NAFLD emerged as a major risk factor for end-stage liver disease, as mortality increases exponentially with fibrosis progression [29]. In the absence of an established pharmacological therapy, lifestyle modification including dietary adjustments is key for preventing excess liver-related mortality [6]. After 12 weeks, about 1/3 of the participants normalized their HCL (criterion < 5.6%) [1]. Our approach shows promising results in reducing both HCL and liver fibrosis in patients with diabetes-related NAFLD. This is particularly relevant for people with newly diagnosed type 2 diabetes, who exhibit a rise in HCL during the early course of disease, likely resulting from enlarging adipose tissue volume and insulin resistance in the face of impaired hepatic mitochondrial adaptation [30]. Reduced energy availability possibly facilitated a drop in thyroid-stimulating hormone [31]. Patients also improved diastolic BP and lowered HR, rendering subsequent cardio-metabolic protection to these patients at elevated risk of cardiovascular morbidity and mortality [32]. Participants did not change their physical activity behavior during the intervention as evidenced by a constant step count of about 7000 steps/day. This is comparable with estimates from other studies evaluating similar patient cohorts, such as adults with impaired glucose tolerance or overt diabetes [33, 34].

The strength of our study results from the comprehensive metabolic characterization using gold-standard methodology [18]. Telephone coaching is potentially suitable for patients in remote areas not served by specialized centers to improve diabetes self-management. However, the long-term costs and  sustainability of this intervention remain to be investigated. This study did not include a control group with standard care as it represents a feasibility study testing for the first time the viability and efficacy of this app-based approach in patients with type 2 diabetes. Furthermore, it is not possible to differentiate between the effects of the individual components of the approach, and the results must be interpreted in the context of the multifaceted intervention (low-calorie diet in combination with app-based educational support and photographic dietary diary with weekly coaching). As with any dietary intervention study, a possible rebound-effect after study completion cannot be excluded. Participants were instructed to continue an isocaloric diet in order maintain their post-intervention body weight, yet the lack of long-term monitoring after the intervention constitutes a limitation of the study.

Taken together, this study shows that an approach combining a real food-based low-calorie diet supported by an app-guided digital education program for patients with diabetes results in clinically relevant reductions in body weight, fat mass, glycemic control and indicators of NAFLD. This approach represents a promising tool for managing patients with type 2 diabetes.

## Data Availability

The data sets generated and/or analyzed during the current study are not publicly available, since they are subject to national data protection laws and restrictions imposed by the ethics committee to ensure data privacy of the study participants. However, they can be applied for through an individual project agreement with the principal investigator of the German Diabetes Study.

## References

[1] European Association for the Study of the L (2016). EASL-EASD-EASO clinical practice guidelines for the management of non-alcoholic fatty liver disease. Obes Facts.

[2] Roden M, Shulman GI (2019). The integrative biology of type 2 diabetes. Nature.

[3] Hernandez EA, Kahl S, Seelig A, Begovatz P, Irmler M, Kupriyanova Y, Nowotny B, Nowotny P, Herder C, Barosa C, Carvalho F, Rozman J, Neschen S, Jones JG, Beckers J, de Angelis MH, Roden M (2017). Acute dietary fat intake initiates alterations in energy metabolism and insulin resistance. J Clin Investig.

[4] Zaharia OP, Strassburger K, Strom A, Bonhof GJ, Karusheva Y, Antoniou S, Bodis K, Markgraf DF, Burkart V, Mussig K, Hwang JH, Asplund O, Groop L, Ahlqvist E, Seissler J, Nawroth P, Kopf S, Schmid SM, Stumvoll M, Pfeiffer AFH, Kabisch S, Tselmin S, Haring HU, Ziegler D, Kuss O, Szendroedi J, Roden M, German Diabetes Study G (2019) Risk of diabetes-associated diseases in subgroups of patients with recent-onset diabetes: a 5-year follow-up study. Lancet Diabetes Endocrinol 7 (9):684-694. doi:10.1016/S2213-8587(19)30187-1 10.1016/S2213-8587(19)30187-1 31345776

[5] Petersen KF, Dufour S, Befroy D, Lehrke M, Hendler RE, Shulman GI (2005). Reversal of nonalcoholic hepatic steatosis, hepatic insulin resistance, and hyperglycemia by moderate weight reduction in patients with type 2 diabetes. Diabetes.

[6] El-Agroudy NN, Kurzbach A, Rodionov RN, O'Sullivan J, Roden M, Birkenfeld AL, Pesta DH (2019). Are lifestyle therapies effective for NAFLD treatment?. Trends Endocrinol Metab.

[7] Evert AB, Dennison M, Gardner CD, Garvey WT, Lau KHK, MacLeod J, Mitri J, Pereira RF, Rawlings K, Robinson S, Saslow L, Uelmen S, Urbanski PB, Yancy WS (2019). Nutrition therapy for adults with diabetes or prediabetes: a consensus report. Diabetes Care.

[8] Obesity Management for the Treatment of Type 2 Diabetes: Standards of Medical Care in Diabetes-2021 (2021). Diabetes Care 44 (Suppl 1):S100-s110. doi: 10.2337/dc21-S008 10.2337/dc21-S008 33298419

[9] George ES, Forsyth A, Itsiopoulos C, Nicoll AJ, Ryan M, Sood S, Roberts Stuart K, Tierney AC (2018). Practical dietary recommendations for the prevention and management of nonalcoholic fatty liver disease in adults. Adv Nutr.

[10] Miller EF (2020). Nutrition management strategies for nonalcoholic fatty liver disease: treatment and prevention. Clin Liv Dis.

[11] Romero-Gómez M, Zelber-Sagi S, Trenell M (2017). Treatment of NAFLD with diet, physical activity and exercise. J Hepatol.

[12] Lean ME, Leslie WS, Barnes AC, Brosnahan N, Thom G, McCombie L, Peters C, Zhyzhneuskaya S, Al-Mrabeh A, Hollingsworth KG, Rodrigues AM, Rehackova L, Adamson AJ, Sniehotta FF, Mathers JC, Ross HM, McIlvenna Y, Stefanetti R, Trenell M, Welsh P, Kean S, Ford I, McConnachie A, Sattar N, Taylor R (2018). Primary care-led weight management for remission of type 2 diabetes (DiRECT): an open-label, cluster-randomised trial. Lancet.

[13] Taylor R, Al-Mrabeh A, Zhyzhneuskaya S, Peters C, Barnes AC, Aribisala BS, Hollingsworth KG, Mathers JC, Sattar N, Lean MEJ (2018) Remission of human type 2 diabetes requires decrease in liver and pancreas fat content but is dependent upon capacity for beta cell recovery. Cell Metab 28 (4):547–556 e543. doi:10.1016/j.cmet.2018.07.003 10.1016/j.cmet.2018.07.003 30078554

[14] Wharton S, Lau DCW, Vallis M, Sharma AM, Biertho L, Campbell-Scherer D, Adamo K, Alberga A, Bell R, Boulé N, Boyling E, Brown J, Calam B, Clarke C, Crowshoe L, Divalentino D, Forhan M, Freedhoff Y, Gagner M, Glazer S, Grand C, Green M, Hahn M, Hawa R, Henderson R, Hong D, Hung P, Janssen I, Jacklin K, Johnson-Stoklossa C, Kemp A, Kirk S, Kuk J, Langlois MF, Lear S, McInnes A, Macklin D, Naji L, Manjoo P, Morin MP, Nerenberg K, Patton I, Pedersen S, Pereira L, Piccinini-Vallis H, Poddar M, Poirier P, Prud'homme D, Salas XR, Rueda-Clausen C, Russell-Mayhew S, Shiau J, Sherifali D, Sievenpiper J, Sockalingam S, Taylor V, Toth E, Twells L, Tytus R, Walji S, Walker L, Wicklum S (2020) Obesity in adults: a clinical practice guideline. CMAJ 192 (31):E875-e891. doi:10.1503/cmaj.191707 10.1503/cmaj.191707 PMC7828878 32753461

[15] Dounavi K, Tsoumani O (2019). Mobile health applications in weight management: a systematic literature review. Am J Prev Med.

[16] Ghelani DP, Moran LJ, Johnson C, Mousa A, Naderpoor N (2020). Mobile apps for weight management: a review of the latest evidence to inform practice. Front Endocrinol (Lausanne).

[17] Holzmann SL, Holzapfel C (2019). A scientific overview of smartphone applications and electronic devices for weight management in adults. J Pers Med.

[18] Szendroedi J, Saxena A, Weber KS, Strassburger K, Herder C, Burkart V, Nowotny B, Icks A, Kuss O, Ziegler D, Al-Hasani H, Mussig K, Roden M, Group GDS (2016) Cohort profile: the German diabetes study (GDS). Cardiovasc Diabetol 15:59. doi:10.1186/s12933-016-0374-9 10.1186/s12933-016-0374-9 PMC4823856 27053136

[19] Cheyette C, Balolia Y, Francis V, Callaghan S, Turner F (2017) Carbs & cals very low calorie recipes & meal plans: lose weight, improve blood sugar levels and reverse type 2 diabetes. chello publishing

[20] Besser REJ, Shields BM, Casas R, Hattersley AT, Ludvigsson J (2013). Lessons from the mixed-meal tolerance test: use of 90-min and fasting C-peptide in pediatric diabetes. Diabetes Care.

[21] Laufs A, Livingstone R, Nowotny B, Nowotny P, Wickrath F, Giani G, Bunke J, Roden M, Hwang JH (2014). Quantitative liver 31P magnetic resonance spectroscopy at 3T on a clinical scanner. Magn Reson Med.

[22] Cassinotto C, Boursier J, de Lédinghen V, Lebigot J, Lapuyade B, Cales P, Hiriart J-B, Michalak S, Bail BL, Cartier V, Mouries A, Oberti F, Fouchard-Hubert I, Vergniol J, Aubé C (2016). Liver stiffness in nonalcoholic fatty liver disease: a comparison of supersonic shear imaging, FibroScan, and ARFI with liver biopsy. Hepatology.

[23] Zaharia OP, Strassburger K, Knebel B, Kupriyanova Y, Karusheva Y, Wolkersdorfer M, Bódis K, Markgraf DF, Burkart V, Hwang J-H, Kotzka J, Al-Hasani H, Szendroedi J, Roden M (2020). Role of patatin-like phospholipase domain-containing 3 gene for hepatic lipid content and insulin resistance in diabetes. Diabetes Care.

[24] Obesity Management for the Treatment of Type 2 diabetes: standards of medical care in diabetes-2019 (2019). Diabetes care 42 (Suppl 1):S81-s89. doi:10.2337/dc19-S008 10.2337/dc19-S008 30559234

[25] Chavez S, Fedele D, Guo Y, Bernier A, Smith M, Warnick J, Modave F (2017). Mobile apps for the management of diabetes. Diabetes Care.

[26] Pafili K, Roden M (2020) Nonalcoholic fatty liver disease (NAFLD) from pathogenesis to treatment concepts in humans. Mol Metab:101122. doi:10.1016/j.molmet.2020.101122 10.1016/j.molmet.2020.101122 PMC8324683 33220492

[27] Koopman KE, Caan MW, Nederveen AJ, Pels A, Ackermans MT, Fliers E, la Fleur SE, Serlie MJ (2014). Hypercaloric diets with increased meal frequency, but not meal size, increase intrahepatic triglycerides: a randomized controlled trial. Hepatology.

[28] Cotter TG, Rinella M (2020). Nonalcoholic fatty liver disease 2020: the state of the disease. Gastroenterology.

[29] Dulai PS, Singh S, Patel J, Soni M, Prokop LJ, Younossi Z, Sebastiani G, Ekstedt M, Hagstrom H, Nasr P, Stal P, Wong VW, Kechagias S, Hultcrantz R, Loomba R (2017). Increased risk of mortality by fibrosis stage in nonalcoholic fatty liver disease: Systematic review and meta-analysis. Hepatology.

[30] Kupriyanova Y, Zaharia OP, Bobrov P, Karusheva Y, Burkart V, Szendroedi J, Hwang JH, Roden M (2020). Early changes in hepatic energy metabolism and lipid content in recent-onset type 1 and 2 diabetes mellitus. J Hepatol.

[31] Aeberli I, Jung A, Murer SB, Wildhaber J, Wildhaber-Brooks J, Knopfli BH, Zimmermann MB (2010). During rapid weight loss in obese children, reductions in TSH predict improvements in insulin sensitivity independent of changes in body weight or fat. J Clin Endocrinol Metab.

[32] Böhm M, Schumacher H, Teo KK, Lonn EM, Mahfoud F, Ukena C, Mann JFE, Mancia G, Redon J, Schmieder RE, Sliwa K, Marx N, Weber MA, Williams B, Yusuf S (2020). Resting heart rate and cardiovascular outcomes in diabetic and non-diabetic individuals at high cardiovascular risk analysis from the ONTARGET/TRANSCEND trials. Eur Heart J.

[33] Tudor-Locke C, Bell RC, Myers AM, Harris SB, Ecclestone NA, Lauzon N, Rodger NW (2004). Controlled outcome evaluation of the First Step Program: a daily physical activity intervention for individuals with type II diabetes. Int J Obes Relat Metab Disord.

[34] Huffman KM, Sun J-L, Thomas L, Bales CW, Califf RM, Yates T, Davies MJ, Holman RR, McMurray JJV, Bethel MA, Tuomilehto J, Haffner SM, Kraus WE (2014) Impact of baseline physical activity and diet behavior on metabolic syndrome in a pharmaceutical trial: results from NAVIGATOR. Metabolism: clinical and experimental 63 (4):554–561. doi:10.1016/j.metabol.2014.01.002 10.1016/j.metabol.2014.01.002 PMC4103164 24559843

